# Designing an mHealth application for informal carers concerning the management of behavioural and psychological symptoms of dementia: a need analysis survey

**DOI:** 10.1186/s12913-024-11273-9

**Published:** 2024-08-14

**Authors:** Thilanka Jagoda, Samath D. Dharmaratne, Sarath Rathnayake

**Affiliations:** 1 Department of Community Medicine, Faculty of Medicine, University of Peradeniya, Peradeniya, Sri Lanka; 2 Department of Nursing & Midwifery, Faculty of Allied Health Sciences, University of Sri Jayewardenepura, Nugegoda, Sri Lanka; 3 Postgraduate Institute of Medical Sciences, University of Peradeniya, Peradeniya, Sri Lanka; 4 Department of Nursing, Faculty of Allied Health Sciences, University of Peradeniya, Peradeniya, Sri Lanka

**Keywords:** Behavioural and psychological symptoms of dementia, Dementia, Informal carers, mHealth applications, mHealth information seeking

## Abstract

**Background:**

Informal carers face difficulties and challenges when dealing with the behavioural and psychological symptoms of dementia (BPSD) of their family members or friends residing at home. Mobile health (mHealth) applications are promising for educating and supporting carers. However, needs analysis studies have not been conducted in Sri Lanka to develop mHealth applications for informal carers of people with dementia.

**Aims:**

To explore the needs to design an mHealth application for informal carers of people with dementia concerning the management of BPSD.

**Method:**

An exploratory cross-sectional survey was conducted among a convenience sample (*N* = 203) of informal carers as a part of developing an mHealth application. Family members, relatives, or friends who lived with people with dementia and provided care on an unpaid basis for more than three months were included. The questionnaire included sociodemographic data, carer-rated prevalence and severity of BPSD, and informal carers’ knowledge of dementia, carer burden, information-seeking sources, availability of smartphones, mHealth information seeking and perception of mHealth information seeking related to managing BPSD. Descriptive analysis and inferential tests were performed.

**Results:**

Informal carers of people with dementia were predominantly female (70.4%), and 64% showed low knowledge of dementia. Of the participants, 35% reported a high carer burden, 53.7% reported a low burden, and only 11.3% reported no carer burden. Most of their care recipients (97%) had at least one BPSD. The prevalence and severity of BPSD were significantly and positively correlated with the carer burden. The participants’ main source of information was health professionals. Most of them owned smartphones (63.5%), but none used mHealth applications for dementia-related information seeking. Approximately half of the respondents were ready to spend time (52.7%) and money (46.8%) on mHealth information seeking. Perceived mHealth usefulness was significantly associated with dementia knowledge, smartphone ownership, and readiness to spend time and money on mHealth information seeking.

**Conclusion:**

Informal carers of people with dementia were affected by BPSD in their care recipients. This study explored carers’ educational needs concerning dementia, BPSD, and carer burden. Informal carers could adapt mHealth for dementia-related information seeking. Their unmet needs in managing BPSD should be explored.

**Supplementary Information:**

The online version contains supplementary material available at 10.1186/s12913-024-11273-9.

## Background

Dementia is a global public health concern and is common in old age. Although the typical feature of dementia is cognitive changes, behavioural and psychological symptoms of dementia (BPSD) are widespread clusters of manifestations [1, 2] and appear at the early stage of the dementia trajectory [3]. These symptoms include psychotic (e.g., hallucinations, delusions), affective (e.g., anxiety, agitation, irritability, depression), and behavioural (e.g., disinhibition, euphoria, apathy, aberrant motor behaviours) manifestations [1]. BPSD can lead to negative consequences for people affected with dementia, such as distress, misuse of medication, increased mortality, and long-term hospitalisation [4, 5].

Informal carers, especially family members, provide care for most community-dwelling people with dementia. Informal carers are mainly co-residents, for example, family members or, to a lesser extent, individuals not living in the same household, such as neighbours or friends [6]. They experience negative psychological consequences related to managing the BPSD of their loved ones [2, 7]. This situation leads to the development of strain, burden, frustration and depression among carers [2, 8]. The difficulties associated with caring and individualised needs concerning BPSD have been shown to escalate the importance of fulfilling informal carers’ educational and supportive information needs [8, 9].

Adapting digital approaches, including providing health care for people with dementia, is an emerging trend in community-based health interventions [10, 11]. A mobile health (mHealth) application refers to a health-related piece of software designed to improve people’s health via several operations and designs running on mobile devices, such as smartphones, tablet phones and smartwatches [12]. Recent literature reports that mHealth applications are used in healthcare for different purposes, for example, the prevention of diseases, promotion of a healthy lifestyle, diagnosis, health education, self-monitoring, and motivating health-related goal achievements [13, 14]. The features of mHealth applications include navigation, finding healthcare professionals and facilities, reminders (e.g., medication reminders), notifications, tracking the changes in diseases, and chatting, texting and communicating with family, friends and social groups [13, 14]. It is a widely accepted digital health approach, and mHealth applications were developed for informal carers of individuals with dementia concerning social and indirect support, health education, adjusting the activities of daily living, and technology-based monitoring of carers and their care recipients [15–17]. However, a recent systematic review revealed that limited evidence-based mHealth applications have been developed to educate or support informal carers of people with dementia to provide them with information to manage BPSD [18].

The development of an mHealth application is a complex process, and mobile health application developers apply human-centred design (known as user-centred design) to improve effectiveness, usability and user acceptance [19, 20]. Steps in developing an mHealth application include needs assessment, design and development, and evaluation [19, 21]. The purpose of a need assessment phase is to identify the desires or needs of end users to develop user-friendly and acceptable mHealth applications [22].

Sri Lanka is a low- and middle-income country where a high prevalence of dementia is predicted due to the sharp increase in the ageing population [23, 24]. In Sri Lanka, the all-age (crude) dementia incidence rate is 84.3 per 100,000, and the crude prevalence of dementia is 0.6% [25]. According to the Ministry of Health [26], the total registered population of people with dementia was 397 in the Colombo District, where the study was conducted. A recent scoping review related to dementia care in Sri Lanka reported that there are no properly implemented screening programmes to detect individuals with dementia in Sri Lanka [27]. In Sri Lanka, different approaches are used to diagnose dementia clinically, for example, according to the criteria given by the Diagnostic and Statistical Manual of Mental Disorders (DSM IV), International Classification of Diseases (ICD) 10, and National Institute of Neurological and Communicative Diseases and Stroke/Alzheimer’s Disease and Related Disorders Association (NINCDS-ADRDA) [27]. Moreover, cognitive and neuropsychological tests (Cambridge Cognitive Score, Montreal Cognitive Assessment, Neuropsychiatric Inventory, Clinical Dementia Rating Scale), blood and cerebrospinal fluid investigations, brain imaging (computed tomography and magnetic resonance imaging), and interviews with individuals were also used to diagnose this condition [25, 27]. The literature suggests that the prevalence of BPSD is high among people with dementia in Sri Lanka [28, 29], and BPSD are a primary reason that carers seek help from the health sector [29]. However, a lack of awareness of dementia was reported among family carers of people with dementia [29], while their knowledge of dementia was not studied. The literature indicates that informal carers’ knowledge of dementia facilitates their caring role [8]. Approximately 75% of informal carers of patients with dementia in Sri Lanka have experienced a mild to severe carer burden [30]. A systematic review revealed that carer burden was associated with BPSD, poor self-care ability and lack of supportive systems [31]. Therefore, multidimensional interventions, mainly focusing on dementia and the management of BPSD, should be implemented for informal carers in Sri Lanka to provide educational and supportive information.

There are no reported studies concerning the development of mHealth applications for informal carers of people with dementia in Sri Lanka [18]. To the authors’ knowledge, evidence-based data were unavailable for the researchers to understand how mHealth interventions would be accepted by informal carers of people with dementia. Information seeking models, for example, the comprehensive model of information seeking, are helpful in assessing how people use and accept mHealth applications for their health-related problems [32, 33]. This model states that individuals’ decision to search for information is based on their desires to find information, information seeking behaviours, and the attributes of the information sources [32, 33]. These factors can be assessed in the need analysis phase of developing an information source such as an mHealth application.

### Aims

This explorative survey study was part of a large study that aimed to develop an mHealth application for informal carers of people with dementia. This study aimed to explore informal carers’ knowledge of dementia, carer burden, information sources, and previous experiences and perceptions of mHealth information seeking. In addition, carer-reported prevalence and severity of BPSD among their care recipients were exploerd.

### Research questions

1. What is the knowledge of dementia among informal carers of people with dementia?
2. What is the carer-reported prevalence and severity of BPSD in people with dementia?
3. What is the carer burden of informal carers of people with dementia?
4. What is the use of mHealth applications in information seeking among informal carers of people with dementia?
5. What is the perception among informal carers of people with dementia about mHealth application information seeking in managing BPSD?

## Methodology

### Study design

An exploratory cross-sectional survey was conducted to explore the needs related to developing an mHealth application for informal carers of people with dementia to address the requirements related to managing BPSD.

### Study setting

The present multicenter study included informal carers from two psychogeriatric clinics at the National Institute of Mental Health, Sri Lanka, and the National Hospital of Sri Lanka (NHSL) (Colombo); a neurology clinic at the NHSL (Colombo); and a psychiatric clinic at the Colombo East Base Hospital, Sri Lanka.

### Participants

The study population included informal carers over 18 years who delivered care for individuals diagnosed with any subtype of dementia. Family members, relatives, or friends who lived in the same household and provided care for an individual with dementia on an unpaid basis for more than three months at the time of recruitment were included. Carers who did not live with their care recipients in the same household were excluded since they may not have lived full-time with a person with dementia and may not have experienced some BPSD being prominent in the evening or nighttime. Carers who could not communicate in the Sinhala language and who had communication difficulties, such as hearing impairment and disorientation to time and place, were excluded.

### Sampling and sample size

The population of Colombo was 2,477,922 in 2022, and 219,038 of whom were over 65 years old [34]. This study used the Yamane formula to calculate the sample size [35]. Using the Annual Health Bulletin of the Ministry of Health data, the total population of people with dementia in Colombo was considered to be 397 [26]. With the addition of 10% nonresponses (*n* = 20), the minimum required sample was 219 carers. A convenience sample of informal carers (*N* = 219) of people with dementia was invited.

### Measures

An interviewer-administered questionnaire was used for data collection. The study variables were chosen based on previous researches [36, 37] and the concept of information seeking [32, 33]. The questionnaire was pretested with ten informal carers, and necessary changes were made before administering it. The study variables were arranged into eight sections. Section one included the sociodemographic data of carers: age, sex, educational attainment, marital status, work status, duration of caring, and relationship with care recipients [37–40]. The sociodemographic data of the care recipients included age, type of dementia and comorbidities [39, 40].

Section two assessed informal carers’ knowledge of dementia using the 25-item Dementia Knowledge Assessment Scale (DKAS) [41]. This 5-point Likert scale comprises four subscales: “*causes and characteristics*,* communication and behaviour*,* care considerations and risks and health promotion*”. This study used the Sinhala-translated version of the DKAS (available at https://www.utas.edu.au/wicking/research/distinct-projects/dementia-knowledge-assessment-scale-registration). A Cronbach’s alpha of 0.71 demonstrated the acceptable reliability of the DKAS for the present study participants.

In section three, the prevalence of BPSD among care recipients with dementia was assessed using the 12-item Neuropsychiatric Inventory Questionnaire (NPI-Q) (α = 0.88) [42]. The prevalence and severity of BPSD were assessed. With prior permission, translation and cultural adaptation of the NPI-Q were performed following the copyright authors’ guidelines [43]. The psychometric properties of the translated Sinhala version (NPI_Q_Sinhala) were assessed by checking the content validity index (CVI), face validity and Cronbach’s alpha. Cronbach’s alpha was acceptable (0.79) [44].

In section four, the Zarit Burden Interview (ZBI) (a screening version with 4 items) (α = 0.79) was used to assess the carer burden [45]. The correlation between the 22-item version and the screening versions was reported to be between 0.83 and 0.93 [45]. The ZBI is a five-point Likert scale. A score of eight or more indicates the cut-off point for a high carer burden [45]. This study used the Sinhala version of the ZBI [30]. The Cronbach’s alpha of the ZBI scale (0.87) was acceptable in the present study.

Section five assessed the frequency of dementia-related information seeking from different sources in the past three months using a Likert scale (*never*,* sometimes*,* often*). The information sources included healthcare professionals (e.g., doctors, nurses, and other healthcare professionals), family or friends, the internet, and smartphones. Section six assessed whether carers owned smart devices such as smartphones, tablets, iPads and smartwatches [37]. The experience of using smartphone technology and the frequency and time spent on smartphones were assessed in section seven [37].

Section eight assessed carers’ perceptions of smartphone technology for information seeking regarding readiness, usefulness and confidence in using the mHealth application. Two questions assessed perceived readiness: (i) “I have time to learn it to find health information from a smartphone if I have a smartphone” (*would not learn*,* might or might not learn*,* definitely learn)* and (ii) “I can spend money to find health-related information using a smartphone” (*would not spend*,* might or might not spend*,* definitely spend)* [46]. Perceived usefulness was assessed using a 5-point Likert scale: “Finding health information through a smartphone would be *“very useless*,* useless*,* somewhat useful*,* very useful*,* no idea”* [46]. Moreover, confidence in mHealth application information seeking was assessed: *“Finding health information through a smartphone would be very inconvenient*,* inconvenient*,* convenient*,* very convenient*,* no idea”* [46, 47].

### Data collection

The study was conducted between August 2022 and February 2023. This study was approved by the National Institute of Mental Health, Sri Lanka; NHSL (Colombo); and Colombo East Base Hospital, Sri Lanka. The interviews were conducted at the clinic premises. During data collection, the nurse-in-charge/nurses of the clinics introduced the researcher to carers before the data collection. In addition, carers’ telephone numbers were collected from the clinics, and they were invited over the phone. Consequently, some interviews were conducted at the participants’ homes since they faced difficulties attending the clinics. The participants were provided with written information, and written informed consent was obtained before data collection. The first researcher (TJ) and two trained registered nurses collected the data. Interviewer-administered interviews were conducted to fill out the questionnaires used in this study. The duration for completing the questionnaire was approximately 15–20 min.

### Statistical analysis

The Statistical Package for Social Sciences software (SPSS) version 25 was utilised for the data analysis. The first researcher manually checked the data for accuracy and completeness (TJ), followed by performing frequency tests in SPSS. Data cleaning was conducted before the analysis. First, listwise deletion was performed. Thus, individual cases from the data set were excluded if more than 5% of the values were missing. Second, single value imputation using means was used to replace missing data that were less than 5% within a row; as a result, 1–3 missing values of a few items were filled using means. Normality was assessed for the scale data by the Shapiro‒Wilk test, for which *p* > .05 indicated statistical significance. The DKAS data showed a normal distribution; however, the ZBI and perceived severity of BPSD data were not normally distributed.

The following descriptive statistics were computed: (i) frequencies and percentages for categorical variables (e.g., sociodemographic information; item-level results of the questionnaire; and binary outcomes of knowledge, burden, the perception of using smartphone technology for information seeking); and (ii) means and standard deviations (e.g., age and normally distributed data, including the DKAS and subscales of the DKAS) or medians (e.g., nonnormally distributed data, including the NPI-Q and ZBI) for the continuous variables.

Knowledge concerning dementia was transformed into a binary outcome variable using the 60th percentile [48]: *sufficient* or *insufficient knowledge*. The relationships between knowledge scores and sociodemographic groups were analysed using parametric tests: independent t-tests/ANOVA. Moreover, the differences between sociodemographic factor groups and nonnormally distributed data (burden score) were computed using Mann‒Whitney (*U*)/Kruskal‒Wallis (*H*) tests. Spearman correlation (r_s_) was calculated to examine correlations between ordinal variables and nonnormally distributed variables, including carer burden scores and BPSD prevalence/severity scores. Pearson’s chi-square test was computed to explore the associations of the binary outcome of the usefulness of mHealth health information seeking with categorical variables, including knowledge of dementia, the perceived severity of BPSD, carer burden, smartphone ownership, readiness to spend time and readiness to spend money on mHealth application information seeking, and the sociodemographic details of the carers. The level of significance (p value) was set at 0.05.

## Results

### Demographic and clinical characteristics

Among the 219 invited informal carers, 203 responded, representing 92.7% of the response rate (*n* = 9 missing data; *n* = 7 refused to participate). The mean age of the informal carers was 53.04 ± 13.79 years, and the mean age of individuals with dementia was 72.9 ± 9.24 years. Almost all carers (93.1%) were unaware of the subtype of dementia of their care recipients. The sociodemographic information of the carers and care recipient dyads is shown in Table 1.

**Table 1:** Descriptive statistics: sociodemographic infomation of informal carers (*N* = 203) and people with dementia (*N* = 203)

Informal carers		People with dementia	
Characteristic	*n* (%)	Characteristic	*n* (%)
**Age category (years)**		Type of dementia*	
Youth (< 25)	4 (2.0%)	Alzheimer’s disease	4 (2.0%)
Young age (25–44)	56 (27.6%)	Vascular	5 (2.5%)
Middle age (45–59)	70 (34.5%)	Fronto-temporal	1 (0.5%)
Elderly age (60–74)	61 (30.0%)	Other types	4 (2.0%)
Senile age (75–90)	12 (5.9%)	Do not know	189 (93.1%)
**Gender**		**Chronic diseases**	
Male	60 (29.6%)	Diabetic Mellitus	51 (25.1%)
Female	143 (70.4%)	Cardiac diseases	30 (14.8%)
Other	0	Blood pressure	68 (33.5%)
		Respiratory diseases	18 (8.9%)
**Marital status**		Gastrointestinal diseases	5 (2.5%)
Married	171 (84.2%)	Psychiatric diseases	27 (13.3%)
Single	23 (11.3%)	Other diseases	21 (10.3%)
Divorced	3 (1.5%)		
Widowed	5 (2.5%)		
Other	1 (0.5%)		
**Educational attainment**			
No education	1 (0.5%)		
Primary education	11 (5.4%)		
Secondary education	162 (79.8%)		
> secondary education	29 (14.2%)		
**Working status**			
Government sector	19 (9.4%)		
Private sector	39 (19.2%)		
Self-Employee	28 (13.8%)		
Retired	31 (15.3%)		
No employment	84 (41.4%)		
Student	2 (1.0%)		
**Duration of providing care to an individual with dementia**			
Year < 1	49 (24.1%)		
1–5 Years	112 (55.2%)		
6–10 Years	27 (13.3%)		
11–15 Years	3 (1.5%)		
16–20 Years	6 (3.0%)		
21–25 Years	3 (1.5%)		
> 25 Years	3 (1.5%)		
**Relationship with individuals with dementia**			
Husband/ wife	54 (26.6%)		
Sister/ brother	12 (5.9%)		
Mother/ father	115 (56.7%)		
Son/daughter	3 (1.5%)		
Son/daughter in-law	1 (0.5%)		
Grandfather/mother	5 (2.5%)		
Friend	1 (0.5%)		
Other	12 (5.9%)		

*Carer reported types of dementia

### Knowledge of dementia

The respondents obtained an overall mean score of 17.60 ± 7.12 out of 50 in the DKAS, indicating a low level of dementia knowledge. The categorisation of knowledge (low and high levels) revealed that the majority (64%) demonstrated low levels of knowledge. The standardised mean scores for the DKAS and its subscale are reported in Table 2. Supplementary material 1 shows the carers’ item-level knowledge.

**Table 2:** Dementia knowledge and carer burden of informal carers (*N* = 203)

Variable	Scale and scores	Mean ± SD*	Median	Range	Standardised score/ 100
Dementia knowledge	Si-DKAS (0–50 scores)	17.60 ± 7.12		0–33	35.19
	Cause & characteristics subscale (0–14 scores)	3.66 ± 2.68		0–11	26.11
	Communication & behaviour subscale (0–12 scores)	2.92 ± 2.32		0–10	24.30
	Care consideration subscale (0–12 scores)	6.62 ± 3.24		0–12	55.17
	Risk & health promotion subscale (0–12 scores)	4.40 ± 2.45		0–10	36.70
Carer burden	ZBI-04		5	0–16	

Si-DKAS = Sinhala Dementia Knowledge Assessment Scale; ZBI-04 = Screening version of the Zarit Burden Interview

### Carer burden

The median ZBI-04 score was 5 out of 16 (Table 2). In the sample, one-third of respondents (35%) experienced a high level of burden. Approximately half of the respondents (53.7%) reported experiencing a low carer burden, and 11.3% of informal carers had not experienced a carer burden.

### Carer-rated prevalence and severity of BPSD

The descriptive analyses of the prevalence and severity of BPSD in the care recipients of the study participants based on the NPI-Q-Sinhala are displayed in Table 3. The median BPSD was 6 out of 12 (range, 0–12). Most care recipients (97%) had at least one BPSD, and 4% had all BPSD assessed. The most prevalent symptoms were nighttime behaviours (66%), apathy/indifference (64%), agitation (61.1%), and irritability (60.6%), and the least common symptom was elation (approximately 25%). The median BPSD severity score was 11 out of 36 (range, 0–31). The severity of each BPSD is reported in Table 3.

**Table 3:** Descriptive statistics: prevalence and the severity of BPSD in people with dementia (*N* = 203)

Type of BPSD	Prevalence *n* (%)	Severity of each BPSD		
		Noticeable *n* (%)	Significant *n* (%)	Dramatic *n* (%)
1. Delusions	102 (50.2%)	24 (11.82%)	47 (23.15%)	31 (15.27%)
2. Hallucinations	100 (49.3%)	31 (15.27%)	48 (23.65%)	21 (10.34%)
3. Agitation/Aggression	124 (61.1%)	50 (24.63%)	45 (22.17%)	29 (14.29%)
4. Depression/Dysphoria	98 (48.3%)	47 (23.15%)	38 (18.72%)	13 (6.40%)
5. Anxiety	100 (49.3%)	39 (19.21%)	46 (22.66%)	15 (7.39%)
6. Elation/Euphoria	51 (25.1%)	26 (12.81%)	22 (10.84%)	3 (1.48%)
7. Apathy/Indifference	130 (64%)	49 (24.14%)	42 (20.69%)	39 (19.21%)
8. Disinhibition	108 (53.2%)	34 (16.75%)	47 (23.15%)	27 (13.30%)
9. Irritability/Lability	123 (60.6%)	45 (22.17%)	57 (28.08%)	22 (10.84%)
10. Motor Disturbance	111 (54.7%)	38 (18.72%)	42 (20.69%)	31 (15.27%)
11. Night-time Behaviours	134 (66%)	40 (19.70%)	54 (26.60%)	39 (19.21%)
12. Appetite/Eating changes	97 (47.8%)	48 (23.65%)	32 (15.76%)	17 (8.37%)

BPSD = Behavioural and Psychological Symptoms of Dementia

### Sources of dementia information seeking

Significantly, a few informal carers reported often having information on dementia from the internet (15.3%) and smartphones (9.4%). Their primary information sources were doctors, nurses, and other healthcare professionals (73.9%). Table 4 shows the frequency of information obtained through different sources.

**Table 4:** Descriptive statistics: factors influencing the acceptance of mHealth application among informal carers (*N* = 203)

Variable		Prevalence (n, %)				
Sources of dementia information-seeking in the last 3 months	Health professionals	Never 53 (26.1%)	Rarely 67 (33%)	Often 83 (40.9%)		
	Family, friends	Never 125 (61.6%)	Rarely 45 (22.2%)	Often 33 (16.3%)		
	Internet	Never 147 (72.4%)	Rarely 25 (12.3%)	Often 31 (15.3%)		
	Smart mobile phone/ etc.	Never 168 (82.8%)	Rarely 16 (7.9%)	Often 19 (9.4%)		
Experience with smartphone use in searching for health-related information	Smartphone ownership	Yes 129 (63.5%)	No 74 (36.5%)			
	Use any mobile application	Yes 84 (41.4%)	No 119 (58.6%)			
	Use of mHealth applications	Yes 30 (14.8%)	No 173 (85.2%)			
	Frequency of using any mobile applications	Daily 58 (28.6%)	1–3 days a week 15 (7.4%)	Several times a month 10 (4.9%)	Very rarely in a year 1 (0.5%)	Not applicable * 119 (58.6%)
	Duration of using a mobile application in one session	< 30 min 50 (24.6%)	30–60 min 17 (8.4%)	> 1 h 17 (8.4%)	Not applicable* 119 (58.6%)	
Readiness towards the mHealth application information seeking	“I have time to learn it to find health information from a mobile phone if I have a mobile phone”.	Would not have time 29 (14.29%)	Might or might not have time 67 (33.00%)	Definitely, I have time 107 (52.71%)		
	“I can spend money to find health-related information using a smart mobile phone.”	Would not spend 41 (20.20%)	Might or might not spend 67 (33.00%)	Definitely spend 95 (46.80%)		
Perceived usefulness towards the mHealth application information seeking	“Finding health information through a smart mobile phone would be”.	Very useless 4 (1.97%)	Useless 8 (3.94%)	Somewhat useful 78 (38.42%)	Useful 91 (44.83%)	No idea 22 (10.84%)
Confidence about the mHealth application information seeking	“Finding health information through a smart mobile phone would be”	Very inconvenient 17 (8.37%)	Inconvenient 18 (8.87%)	Convenient 85 (41.87%)	Very convenient 51 (25.12%)	No idea 32 (15.76%)

*Smartphone applications were not used

### Experience in using smartphone applications

Nearly two-thirds of informal carers (63.5%) owned smartphones. Fewer than half of them (41.4%) used different mobile applications, and approximately 15% used mHealth applications, including applications concerning physical fitness, COVID-19, diabetes mellitus, hypertension, food intake, and menstruation. None of them used dementia-related mHealth applications. Table 4 illustrates the use of mobile applications and the frequency and time spent on these applications.

### Perceptions of mHealth application information seeking in managing BPSD

Table 4 presents carers’ perceptions of using smartphone technology for information seeking. Approximately half of the respondents indicated that they could afford the time to learn about mHealth applications (52.7%) and money for mHealth application information seeking (46.8%). The majority perceived that mHealth applications would be useful (83.2%). Approximately two-thirds (67%) of the participants believed that seeking health information on smartphones would be convenient.

### Factors affecting the knowledge and carer burden of informal carers

Table 5 presents group differences in knowledge scores based on sociodemographic variables. Knowledge score differences were observed based on age category (*p* < .001) (the highest score in those aged less than 25), educational group (*p* = .018) (the highest score in those with more than secondary education), and relationship with their care recipient group (*p* = .031) (the highest score in mothers/fathers).

**Table 5:** Impact of sociodemographic factors of carers on their knowledge of dementia and carer burden

		Knowledge				Carer Burden		
Variable	*n*	Mean	SD	F/ T value	Sig.	Mean Rank	M-WU/K-WH value	Sig.
**Age category (years)**				^1^F(4,198) = 4.861	< 0.001*		^2^4.18	0.383
Youth (< 25)	4	20.00	2.00			48.38		
Young age (25–44)	56	18.91	7.36			97.77		
Middle age (45–59)	70	19.30	7.03			106.81		
Elderly age (60–74)	61	15.11	6.38			103.43		
Senile age (75–90)	12	13.33	6.64			104.33		
**Work status**				^1^F(5,197) = 0.97	0.436		^2^12.84	0.025*
Government worker	19	17.26	8.84			97.97		
Private sector worker	39	19.36	6.89			92.21		
Self-Employee	28	17.43	7.41			132.96		
Retired	31	18.26	5.99			99.47		
No employment	84	16.58	7.14			99.96		
Student	2	21.00	2.83			22.75		
**Marital status**				^1^F(4,198) = 1.60	0.177		^2^2.01	0.733
Married	171	17.67	7.43			103.23		
Single	23	18.70	4.53			95.20		
Divorced	3	18.67	3.79			117.33		
Widowed	5	10.60	3.97			95.90		
Other	1	11.00	.			33.50		
**Relationship with individuals with dementia**				^1^F(7,195) = 2.26	0.031*		^2^4.17	0.760
Husband/ wife	54	15.48	6.68			100.94		
Sister/ brother	12	15.08	5.33			91.63		
Mother/ father	115	18.84	6.95			105.56		
Son/daughter	3	15.67	9.50			88.33		
Son/daughter in-low	1	9.00	.			12.00		
Grandfather/ mother	5	20.40	5.55			78.50		
Friend	1	6.00	.			98.00		
Other	12	18.67	9.47			104.04		
**Duration of caring**				^1^F(6,196) = 0.887	0.887		^2^3.02	0.806
< 1 Year	49	17.29	7.10			99.89		
1–5 Years	112	17.68	7.34			99.92		
6–10 Years	27	17.07	6.67			107.22		
10–15 Years	3	19.67	11.06			149.67		
16–20 Years	6	16.33	7.58			106.92		
21–25 Years	3	20.00	4.00			123.50		
> 25 Years	3	22.33	2.52			88.33		
**Educational attainment**				^1^F(3,199) = 3.43	0.018*		^2^4.85	0.183
No education	1	16.00	.			12.00		
Primary education	11	14.18	7.25			127.50		
Secondary education	162	17.22	7.10			99.94		
> secondary education	29	21.07	6.29			106.91		
**Gender**				^3^T(201)= -0.79	0.413		^4^3620.50	0.079
Male	60	16.98	7.554			90.84		
Female	143	17.85	6.947			106.68		

^1^One-Way ANOVA, ^2^ Kruskal-Wallis Test, ^3^Independent t-test and ^4^Mann-Whitney Test

Statistical significance at *p*-value < 0.05

Burden scores significantly differed among carers in various work situations (*p* = .025) (highest score in the self-employee group) (Table 5). There were no significant differences in burden scores among the remaining sociodemographic variables.

Significant positive correlations between BPSD incidence and carer burden (*r*_*s*_ = 0.376, *p* < .001) and between BPSD severity and carer burden (*r*_*s*_ = 0.407, *p* < .001) were observed (Table 6).

**Table 6:** Spearman correlation: association of prevalence and severity of BPSD with carer burden

Variable	Carer burden	BPSD/ prevalence	BPSD/ severity
Carer burden score	1.000		
BPSD prevalence	0.376^**^	1.000	
BPSD severity	0.407^**^	0.889^**^	1.000

**Correlation is significant at the 0.01 level (2-tailed)

BPSD = Behavioural and Psychological Symptoms of Dementia

### Factors affecting the perceived usefulness of mHealth application information seeking

There was a significant relationship between the perceived usefulness of mHealth application information seeking and carer age (*p* < .001), knowledge of dementia (*p* = .041), educational attainment (*p* < .002), smartphone ownership (*p* < .001), readiness to spend time seeking mHealth application information (*p* < .001), and readiness to spend money seeking mHealth application information (*p* < .001) (Table 7). Table 7 shows that most carers across any age group perceived that mHealth would be useful for information seeking. Moreover, most carers at any educational level perceived that mHealth would be useful for them. Of the participants with sufficient knowledge of dementia (*n* = 130), 80% perceived that mHealth would be useful.

**Table 7:** The Chi-square test: factors affecting the perceived usefulness of mHealth applications

Variable	X^2^	df	*p* value
Age group	18.37	4	0.001*
Gender of carer	2.65	1	0.104
Work situation of carers	8.71	5	0.121
Marital status of carers	7.84	4	0.098
Educational attainment	20.88	6	0.002*
Relationship with individuals with dementia	13.79	7	0.055
Duration of caring	10.12	6	0.120
Knowledge of dementia	4.19	1	0.041*
Carer burden	4.46	2	0.108
Smartphone ownership	32.54	1	0.000*
Time availability to learn mHealth application information-seeking	27.47	1	0.000*
Readiness to spend money for mHealth application information-seeking	27.46	1	0.000*
*Perceived usefulness based on the age category			
**Age category (years)**	**Total no. of carers (n)**	**Perceived as not useful (n)**	**Perceived as useful (n)**
< 30 years	7	1	6
30–39 years	26	2	24
40–49 years	57	4	53
50–59 years	40	6	34
> 60 years	73	21	52
*Perceived usefulness based on the educational attainment			
**Educational attainment**	**Total no. of carers**	**Perceived as not useful (n)**	**Perceived as useful (n)**
No formal education	1	1	0
Up to grade 5	11	2	9
Up to ordinary level	93	25	68
Up to advanced level	69	6	63
Bachelor’s degree	16	0	16
Postgraduate degree	12	0	12
Vocational training	1	0	1
*Perceived usefulness based on knowledge of dementia			
**Level of knowledge**	**Total no. of carers**	**Perceived as not useful (n)**	**Perceived as useful (n)**
Insufficient knowledge	130	27	103
Sufficient knowledge	73	7	66

*Statistical significance at *p* < .05

## Discussion

This study examined the needs related to developing an mHealth application for informal carers of people with dementia. To the best of our knowledge, this is the first need-analysing study for developing an mHealth application focused on informal carers of people living with dementia in Sri Lanka.

The current study explored informal carers’ educational needs in relation to dementia knowledge. They demonstrated insufficient knowledge of dementia. Informal carers in some other countries also demonstrated low levels of dementia. For example, carers in Spain had a low dementia knowledge score (score percentage: less than 50%) [49]. A study in Israel reported that informal carers of patients with Alzheimer’s disease displayed poor levels of knowledge on the prevalence, causes and symptoms of dementia [50]. The current study revealed that carers were not aware of the correct types of dementia. However, they expressed their concern about the comorbidities (other chronic diseases) of their loved ones. The lack of awareness of the subtypes of dementia might be attributed to the lack of information received from healthcare professionals or the lack of information available to the general public. Moreover, among the subscales of the DKAS explored, the carers of the present study showed the lowest knowledge of the *communication and behaviour* subscale. This subscale is directly related to the context of this study, BPSD. Given that the interaction between family carers and care recipients affects BPSD [51], less communication between these parties influences the severity of BPSD among those affected by dementia [52].

Therefore, the initiation of educational interventions for informal carers of people with dementia is timely. Focusing on the general knowledge of dementia and the therapeutic communication skills of informal carers would increase the magnitude of an educational intervention related to BPSD. The literature indicates that informal carers seek information on general knowledge of dementia and diagnosis, the progress of dementia, behavioural changes and caring strategies, available treatments, side effects of medications, and future care [18, 53, 54].

In contrast to the current study, researchers revealed greater dementia knowledge among informal carers of people with dementia in India [55] and the United Kingdom [56]. The greater dementia knowledge of carers might be attributed to the availability of information sources, such as the internet, local events and conferences from reliable sources such as Alzheimer’s Society, and research findings [56]. A recent systematic review confirmed that internet-based sources are a highly utilised means of seeking information for informal carers of people with dementia [57]. Nevertheless, informal carers of people with dementia in Sri Lanka rarely use remote access to information seeking through the internet or mHealth applications. A possible explanation for the low levels of dementia knowledge among informal carers in this study may be related to the lack of familiarity with timely available information sources, such as the internet, despite their high educational level. Furthermore, most of the study participants were from the main capital (Colombo) of Sri Lanka. According to the researchers’ knowledge, most dementia services are concentrated in the Colombo District. Therefore, informal carers in remote districts might face challenges in finding services and acquiring the required knowledge compared to carers in the capital city. Therefore, the findings of the current study suggest a need to address educational information through the internet or smartphone-based interventions.

Notably, caring for BPSD is highly challenging for informal carers. This study revealed that the prevalence of BPSD was high among care recipients. Consistent with previous studies [58, 59], this research revealed that most people with dementia had at least one BPSD. Similar to our findings, a previous systematic review reported common BPSD, such as nighttime behaviours, apathy, agitation/aggression, and irritability, among those affected [60]. Moreover, agitation, repetitive behaviours and apathy were found to affect the daily living activities and social life of individuals with dementia [61]. Consequently, these BPSD need to be prioritised when planning educational interventions.

The current study explored the need for supportive information with reference to carer burden. The literature has reported that a high carer burden among informal carers was associated with BPSD than with cognitive decline [62]. Most of the participants in this study reported a low or high carer burden, while most informal carers were middle-aged females. Similarly, a high percentage of mild to severe carer burden was revealed by a previous study conducted in Sri Lanka [30]. The literature has reported that female carers experienced more carer burdens and adverse health consequences than male carers did [63]. Similar to the current findings, a positive correlation was revealed between the prevalence of BPSD and carer burden [59, 64]. Therefore, carers who are providing care for BPSD need emotional support and information on managing the carer burden [18, 53]. In light of the high information demands of informal carers of people with dementia, difficulties associated with finding supportive information were reported in a previous study [65].

A small percentage of carers in the current study reported no carer burden. A review article reported that carers in Asian countries were highly influenced by their culture; for example, the influence of Buddhist culture might lead to accepting or enduring the caring experience caused by dementia [66]. Future studies can focus on how culture affects carer burden and resilience among carers in Sri Lanka.

Most of the study participants’ dementia-related information sources were health professionals (e.g., nurses and doctors). These carers rarely used mHealth applications to seek information about dementia. Nonetheless, carers in old age, those who had only primary education, or those who had insufficient knowledge of dementia also perceived mHealth applications to be useful. Although there is a paucity of mHealth application usage, we found an association between smartphone ownership and the perceived usefulness of smartphone health information seeking. Knowledge of dementia was also associated with the perceived usefulness of mHealth application information seeking among the present study participants. In line with the results of the current study, the literature revealed that informal carers were willing to accept this time-efficient and easily accessible smartphone/mHealth technology [10, 39]. The literature supports factors affecting mHealth application information seeking behaviours, such as functionality, perceived usefulness, ease of use, mHealth credibility, cost, and advanced educational levels of carers [67, 68]. Previous researchers have reported that informal carers utilise telehealth interventions more frequently if they are familiar with the technology [69]. Seeking information from digital sources, such as mHealth applications and internet-based websites, is highly efficient and cost-effective [10, 69]. Given the perceived usefulness and familiarity with smartphones among a substantial percentage of carers in this study, it can be assumed that they may be ready to use mHealth applications. Despite this, the literature revealed the need to explore cultural appropriateness for health interventions [70].

Our results suggest that informal carers benefit from educational information on dementia and BPSD and supportive information on carer well-being in managing highly prevalent clusters of BPSD in their care recipients. The next phase of this larger project of developing an mHealth application is to explore informal carers’ experiences, carer burdens and information needs in managing BPSD and their preferences regarding the layout and features of a potential mHealth application. The findings of the present study are important for researchers in developing an interview guide for qualitative studies.

## Limitations

The paucity of dementia statistics in the current literature and the possibility of underdiagnosis due to the lack of screening might prevent researchers from identifying the correct dementia population in the Colombo District. Data collection at clinics was challenging due to the difficulties faced by carers in attending the clinics or staying in the clinic with their care recipients for extra time. Consequently, selection bias, that is, the recruitment of a convenience sample of participants from selected clinics in the main capital of Sri Lanka, affects the generalisability of the findings. Moreover, we did not assess any confounding factors, such as the stage of dementia or formal diagnosis of BPSD, affecting the relationships among the study variables, leading to confounding bias. For example, the literature has reported that the stages of dementia are associated with carer burden [71, 72]. In the current study, we were unable to collect data related to the exact stage of dementia, as there were no documented details on the stage of dementia in patient records, adding a limitation to this study. Assessments of the prevalence and severity of BPSD by informal carers may lead to recall bias and subjective interpretations of informal carers.

## Conclusion and recommendation

In summary, informal carers of people living with dementia are primarily middle-aged females, and the prevalence of BPSD is high among their care recipients. The requirements for educational interventions for informal carers of people with dementia were explored in the current study based on their low knowledge of dementia and high carer burden. The educational interventions should focus on general information on dementia, specialised information on BPSD, and maintaining carer well-being. Even though mHealth literacy was at a poor level, most informal carers owned smartphones, and their perception of proposed mHealth information seeking was positive across cohorts of diverse ages and educational levels. Our findings suggest opportunities and challenges for mHealth information seeking among study participants.

The development of sustainable evidence-based mHealth applications is recommended. However, it is necessary to explore informal carers’ specific knowledge of BPSD and unmet information requirements in managing their care recipients’ behavioural and psychological symptoms. Conducting awareness programmes for informal carers of people with dementia with the development of the mHealth application is suggested, given the nonavailability of mHealth applications concerning dementia in the local setting.

### Electronic supplementary material

Below is the link to the electronic supplementary material.

Supplementary Material

## Data Availability

This study is part of an ongoing PhD project of the principal investigator. Therefore, the data will be made available from the corresponding author upon reasonable request.

## Abbreviations

BPSD: Behavioural and Psychological Symptoms of Dementia
CVI: Content Validity Scale
DKAS: Dementia Knowledge Assessment Scale
DSM: Diagnostic and Statistical Manual of Mental Disorders
ICD: International Classification of Diseases
NINCDS-ADRDA: National Institute of Neurological and Communicative Diseases and Stroke/Alzheimer’s Disease and Related Disorders Association
NHSL: National Hospital of Sri Lanka
NPI-Q: Neuropsychiatric Inventory Questionnaire
NPI_Q_Sinhala: Neuropsychiatric Inventory Questionnaire - Sinhala version
ZBI: Zarit Burden Interview
