# Supplementary material for: Designing an mHealth application for informal carers concerning the management of behavioural and psychological symptoms of dementia: a need analysis survey
Source: BMC Health Serv Res. 2024 Aug 14;24:930. doi: 10.1186/s12913-024-11273-9 (PMC11325574; doi:10.1186/s12913-024-11273-9)
Supplement: Supplementary file 1 — Supplementary Material [file 12913_2024_11273_MOESM1_ESM.docx]

**Supplementary Material**

Descriptive statistics: Knowledge of informal carers related to the items of the DKAS (N=203)

| DKAS Subscale | Item | Response | n (%) |
| --- | --- | --- | --- |
| Cause and characteristics | 1. Dementia is a normal part of the ageing process. | Incorrect/do not know responses | 151 (74.4%) |
|  |  | Partially correct response | 10 (4.9%) |
|  |  | Correct response | 42 (20.7%) |
|  | 2. Alzheimer’s disease is the most common form of dementia. | Incorrect/do not know responses | 173 (85.2%) |
|  |  | Partially correct response | 14 (6.9%) |
|  |  | Correct response | 16 (7.9%) |
|  | 3. People can recover from the most common forms of dementia. | Incorrect/do not know responses | 156 (76.8%) |
|  |  | Partially correct response | 18 (8.9%) |
|  |  | Correct response | 29 (14.3%) |
|  | 4. Dementia does not result from physical changes in the brain. | Incorrect/do not know responses | 126 (62.1%) |
|  |  | Partially correct response | 15 (7.4%) |
|  |  | Correct response | 62 (30.5%) |
|  | 5. Planning for end of life care is generally not necessary following a diagnosis of dementia. | Incorrect/do not know responses | 60 (29.6%) |
|  |  | Partially correct response | 20 (9.9%) |
|  |  | Correct response | 123 (60.6%) |
|  | 6. Blood vessel disease (vascular dementia) is the most common form of dementia. | Incorrect/do not know responses | 188 (92.6%) |
|  |  | Partially correct response | 3 (1.5%) |
|  |  | Correct response | 12 (5.9%) |
|  | 7. Blood vessel disease (vascular dementia) is the most common form of dementia. | Incorrect/do not know responses | 147 (72.4%) |
|  |  | Partially correct response | 18 (8.9%) |
|  |  | Correct response | 38 (18.7%) |
| Risks and health promotion | 8. Having high blood pressure increases a person’s risk of developing dementia. | Incorrect/do not know responses | 118 (58.1%) |
|  |  | Partially correct response | 38 (18.7%) |
|  |  | Correct response | 47 (23.2%) |
|  | 9. Maintaining a healthy lifestyle does not reduce the risk of developing the most common forms of dementia. | Incorrect/do not know responses | 104 (51.2%) |
|  |  | Partially correct response | 22 (10.8%) |
|  |  | Correct response | 77 (37.9%) |
|  | 10. Symptoms of depression can be mistaken for symptoms of dementia. | Incorrect/do not know responses | 124 (61.1%) |
|  |  | Partially correct response | 40 (19.7%) |
|  |  | Correct response | 39 (19.2%) |
|  | 11. Exercise is generally beneficial for people experiencing dementia. | Incorrect/do not know responses | 48 (23.6%) |
|  |  | Partially correct response | 32 (15.8%) |
|  |  | Correct response | 123 (60.6%) |
|  | 12. Early diagnosis of dementia does not generally improve quality of life for people experiencing the condition. | Incorrect/do not know responses | 102 (50.2%) |
|  |  | Partially correct response | 24 (11.8%) |
|  |  | Correct response | 77 (37.9%) |
|  | 13. The sudden onset of cognitive problems is characteristic of common forms of dementia. | Incorrect/do not know responses | 194 (95.6%) |
|  |  | Partially correct response | 6 (3.0%) |
|  |  | Correct response | 3 (1.5%) |
| Communication and behaviour | 14. It is impossible to communicate with a person who has advanced dementia. | Incorrect/do not know responses | 153(75.4%) |
|  |  | Partially correct response | 13 (6.4%) |
|  |  | Correct response | 37 (18.2%) |
|  | 15. A person experiencing advanced dementia will not generally respond to changes in their physical environment. | Incorrect/do not know responses | 164 (80.8%) |
|  |  | Partially correct response | 11 (5.4%) |
|  |  | Correct response | 28 (13.8%) |
|  | 16. It is important to correct a person with dementia when they are confused. | Incorrect/do not know responses | 156 (76.8%) |
|  |  | Partially correct response | 18 (8.9%) |
|  |  | Correct response | 29 (14.3%) |
|  | 17. People experiencing advanced dementia often communicate through body language. | Incorrect/do not know responses | 120 (59.1%) |
|  |  | Partially correct response | 33 (16.3%) |
|  |  | Correct response | 50 (24.6%) |
|  | 18. Uncharacteristic behaviours in a person experiencing dementia are generally a response to unmet needs. | Incorrect/do not know responses | 91 (44.8%) |
|  |  | Partially correct response | 41 (20.2%) |
|  |  | Correct response | 71 (35.0%) |
|  | 19. Medications are the most effective way of treating behavioural symptoms of dementia. | Incorrect/do not know responses | 172 (84.7%) |
|  |  | Partially correct response | 16 (7.9%) |
|  |  | Correct response | 15 (7.4%) |
| Care considerations | 20. People experiencing dementia do not generally have problems making decisions. | Incorrect/do not know responses | 75 (36.9%) |
|  |  | Partially correct response | 19 (9.4%) |
|  |  | Correct response | 109 (53.7%) |
|  | 21. Movement is generally affected in the later stages of dementia. | Incorrect/do not know responses | 65 (32.0%) |
|  |  | Partially correct response | 41 (20.2%) |
|  |  | Correct response | 97 (47.8%) |
|  | 22. People with advanced dementia may have difficulty speaking. | Incorrect/do not know responses | 89 (43.8%) |
|  |  | Partially correct response | 43 (21.2%) |
|  |  | Correct response | 71 (35.0%) |
|  | 23. People experiencing dementia often have difficulty learning new skills. | Incorrect/do not know responses | 64 (31.5%) |
|  |  | Partially correct response | 51 (25.1%) |
|  |  | Correct response | 88 (43.3%) |
|  | 24. Difficulty eating and drinking generally occurs in the later stages of dementia. | Incorrect/do not know responses | 110 (54.2%) |
|  |  | Partially correct response | 27 (13.3%) |
|  |  | Correct response | 66 (32.5%) |
|  | 25. Daily care for a person with advanced dementia is effective when it focuses on providing comfort. | Incorrect/do not know responses | 31 (15.3%) |
|  |  | Partially correct response | 43 (21.2%) |
|  |  | Correct response | 129 (63.5%) |

DKAS, Dementia Knowledge Assessment Scale
